# Two new synonyms and a name at new rank of *Oreocharis* Benth. (Gesneriaceae) in China

**DOI:** 10.1186/s40529-025-00485-9

**Published:** 2025-12-16

**Authors:** Xiaokai Xiong, Meijun Li, Zhi Li, Fang Wen, Xinxiang Bai

**Affiliations:** 1 College of Forestry, Guizhou University, Guiyang, Guizhou CN-550025 China; 2 Guangxi Key Laboratory of Plant Conservation and Restoration Ecology in Karst Terrain, Guangxi Institute of Botany, Guangxi Zhuang Autonomous Region and Chinese Academy of Sciences, Guilin, Guangxi Zhuang Autonomous Region CN-541006 China; 3 Gesneriad Committee of China Wild Plant Conservation Association, National Gesneriaceae Germplasm Resources Bank of GXIB, Gesneriad Conservation Center of China (GCCC), Guilin, Guangxi CN-541006 China

**Keywords:** *Oreocharis*, Molecular systematics, Haplotype, *Oreocharis brachypoda*, *Oreocharis wanshanensis*, Synonym

## Abstract

**Background:**

The enlarged concept of*Oreocharis* Benth., as the second largest genus of Gesneriaceae plants in China, has been a focus of attention for Gesneriaceae researchers in recent years. However, after the frequent taxonomic revisions of *Oreocharis*, errors in species publication have become common, leading to confusion in taxonomy. Two taxonomic issues requiring attention were identified during the investigation of the resources of Gesneriaceae in the karst plateau of Guizhou. First, the morphological characteristics of *Oreocharis brachypoda* J.M.Li & Zhi M.Li and *Oreocharis wanshanensis* (S.Z.He) Mich.Möller & A.Weber are difficult to distinguish, and their type localities overlap. Therefore, it is suspected that the same species has been repeatedly published, albeit with different specific epithets. Moreover, the above two species are closely related to the *Oreocharis villosa* (K.Y.Pan) Mich.Möller & A.Weber. Second, there is no essential difference in morphological characteristics between *Oreocharis notochlaena* H.Lév. and *Oreocharis mileensis* (W.T.Wang) Mich.Möller & A.Weber, and the latter should be treated as a synonym of *Oreocharis notochlaena*. In this study, through morphological, molecular phylogenetic, and haplotype network studies, the taxonomic status of the above involved species was explored.

**Results:**

Based on original literature research, field investigation, and morphological comparison, it was determined that the morphological characteristics of *Oreocharis brachypoda* and *O. wanshanensis* are highly consistent, and there are no qualitative characteristics that can effectively distinguish the two, while quantitative characteristics overlap with each other. *O. villosa* differs from the above two by cymes 2-3 branches, 2-3 per plant, each 8-10-flowered, anther thecae divergent, style shorter than ovary. Similarly, it is difficult to distinguish between *O. notochlaena* and *O. mileensis* in morphology. Both the reconstructed phylogenetic relationships and the results of the haplotype network analysis also corroborate the results of the morphological studies.

**Conclusion:**

Based on morphology, geographic distribution patterns, molecular phylogeny, and haplotype networks, the findings on two taxonomic issues are as follows: First, *O. brachypoda* is a duplicate publication of *O. wanshanensis*, and morphologically, it is distinguished from *O. villosa* by cymes unbranched, 1-4-flowered, anther thecae confluent, style and ovary nearly equal in length. Second, *O. mileensis* is a duplicate publication of *O. notochlaena*. According to the regulations and suggestions of the 2018 *International Code of Nomenclature for Algae, Fungi, and Plants (Shenzhen Code)*, it is proposed to treat *O. wanshanensis* as a variant of *O. villosa* and to treat *O. brachypoda* as a synonym of *O. wanshanensis*. At the same time, *O. mileensis* should be treated as a synonym of *O. notochlaena*.

**Supplementary Information:**

The online version contains supplementary material available at 10.1186/s40529-025-00485-9.

## Background

*Oreocharis* Benth. was established by George Bentham (1876). In early taxonomic studies, there were approximately 29 species included in the systematic compilation by Wang et al. (1998) and Li and Wang (2005), of which 28 species and five varieties were distributed in China. Since 2011, based on extensive molecular systematics and morphological studies, *Oreocharis* has been incorporated into the genera *Isometrum* Craib, *Bournea* Oliv., *Dayaoshania* W.T.Wang, *Deinocheilos* W.T.Wang, *Opithandra* B.L.Burtt, *Paraisometrum* W.T.Wang, *Ancylostemon* Craib, *Thamnocharis* W.T.Wang, *Tremacron* Craib, and *Briggsia* Craib (the rosulate taxa) to form *Oreocharis* s.l (Möller et al. 2011a, 2011b; Chen et al. 2014, 2014). Although in 2020, *Bournea* Oliv. was once again split off as an independent genus (Chen et al. 2020), further research indicates that *Bournea* should indeed be reintegrated into *Oreocharis* s.l (Kong et al. 2022). *Oreocharis* s.l. scarcely differ in vegetative habit and fruit characteristics. All are rosette plants with spirally arranged leaves, axillary inflorescences, and capsule loculicidal, usually long and cylindrical, occasionally ovoidal (Möller et al. 2011b). However, *Oreocharis* s.l. exhibits extremely diverse floral characteristics, with corolla shapes including tubular, slender-tubular, tubular-funnel, campanulate, urceolate, and rotate; corolla colors range from white, red, yellow, purple, and multicolored; corolla symmetry ranges from actinomorphic and zygomorphic; and the characteristics of the stamens also show significant variation in terms of their position, adhesion, and fertile quantity. The diverse floral characteristics cover the floral attributes of Subfam. Didymocarpoideae Arn (Jin et al. 2021). At present, there are about 159 species of *Oreocharis* s.l., mainly distributed in most parts of southwestern and southern China, with about 10 species distributed in Vietnam, Myanmar, Japan, and Thailand (Wei et al. 2022; Tran et al. 2023; GRC 2024).

**Fig. 1:**
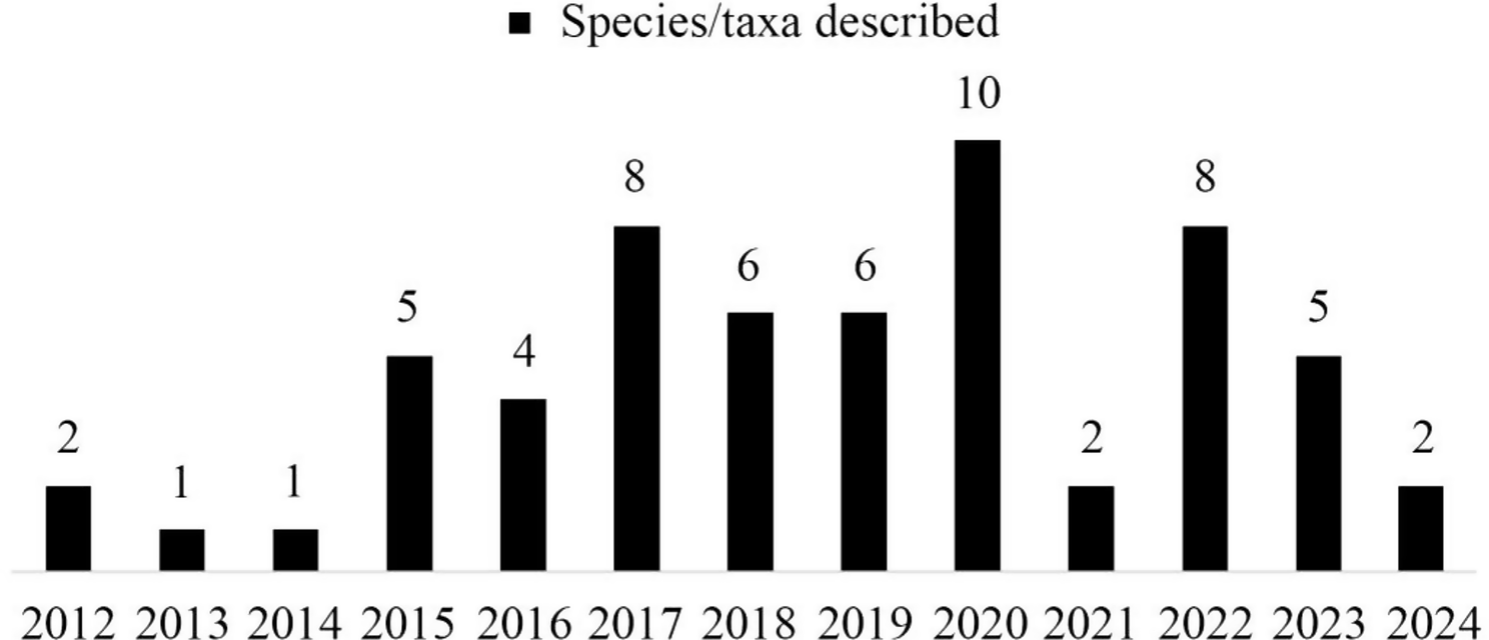
Number of *Oreocharis* species/taxa published since 2011

In recent years, *Oreocharis* s.l. has been a focus of attention for researchers in Gesneriaceae, and many scholars have conducted research on this genus from various perspectives. Kong et al. (2022) conducted a study based on the transcriptomic data of 88% (111/126) of all species in this genus, revealing species diversification dynamics and the possible driving forces of diversity formation. This study provides a strong phylogenetic hypothesis for most species in the genus and lays a solid foundation for further research on species identification and classification. Some supplementary descriptions of early published species morphology have been continuously completed in recent years, such as *O. rhytidophylla* C. Y. Wu ex H. W. Li, *O. dasyantha* Chun, and *O. flavida* Merr (Zhang et al. 2019; Ye 2021). Meanwhile, as researchers have explored previously inaccessible remote areas, new taxa have been continuously described and published. In 2011, Möller et al. (2011) redefined *Oreocharis* s.l. and confirmed 102 species. Since then, up to 2024, 60 new taxa have been published in the genus (Fig. 1). Further morphological and molecular systematics studies have resulted in some species being revised into *Oreocharis* s.l (Chen et al. 2014; Möller et al. 2014; Yang et al. 2020),. making it the second largest genus of Gesneriaceae plants in China, second only to *Primulina* Hance. After the frequent taxonomic revisions of *Oreocharis*, numerous changes have occurred in the systematic position and scientific names of species. In addition, due to differences in the acceptance of different taxonomic systems by scholars, it is relatively easy to make errors in the publication of species, resulting in confusion in taxonomy. At the same time, *Oreocharis* s.l. has high endemism and a long research history, especially the publication of many species by European botanists in the early days. However, due to insufficient field investigation and research, taxonomic problems are relatively common.

In this study, based on extensive investigations of the *Oreocharis* plant resources in the karst plateau of Guizhou over the years, two taxonomic problems requiring attention were identified. First, the morphological characteristics of *O. wanshanensis* (S.Z.He) Mich.Möller & A.Weber and *O. brachypoda* J.M.Li & ZhiM.Li are basically the same, and their type locality overlaps, so they may be duplicate publications of the same species. In April 2003, a scholar discovered a plant of Gesneriaceae in Shamu Cave, Wanshan District, Tongren City, Guizhou Province. After carefully comparing its morphological characteristics, it was identified as a new species of *Isometrum*, named *Isometrum wanshanensis* S.Z.He (He 2006). Subsequently, *Isometrum* was merged into *Oreocharis* s.l., and the scientific name of *Isometrum wanshanensis* was changed to *Oreocharis wanshanensis* (S.Z.He) Mich.Möller & A.Weber. In April 2014, another scholar collected a plant of Gesneriaceae in Jiulong Cave, Bijiang District, Tongren City, and published it as *Oreocharis brachypodus* J.M.Li & Zhi M.Li (Li and Li 2015). Afterward, Cai et al. (2020) pointed out that the part of speech of the suffix “us” in the specific epithet does not match the genus name and corrected it to *Oreocharis brachypoda* J.M.Li & Zhi M.Li. Both of them are accepted by well-known botanical taxonomy name catalog websites such as IPNI (http://www.ipni.org), Tropicos (http://www.tropicos.org), and POWO (https://powo.science.kew.org). However, in the protologue descriptions of the two species, we found that the species’ taxonomic treatment and characteristic descriptions were consistent. After investigating the type localities of the two species, we found that the morphological characteristics were consistent. The straight-line distance between the type localities was only about 20 kilometers (Fig. 12). In previous studies based solely on morphological characteristics, *Oreocharis villosa* (K.Y.Pan) Mich.Möller & A.Weber was identified as a related species to *O. brachypoda* and *O. wanshanensis* (He 2006; Li and Li 2015). *Isometrum villosum* K.Y.Pan was published in 1986 by Kaiyu Pan (1986) based on specimens collected from Shizhu County, Chongqing City. After the establishment of *Oreocharis* s.l., the scientific name for the species was changed to *Oreocharis villosa* (K.Y.Pan) Mich.Möller & A.Weber.

Second, with the rediscovery of *Oreocharis notochlaena* H.Lév. by our group in Zhaosi Village, Huaxi District, and Wangyou Town, Huishui County, Guizhou Province, we found no essential difference in morphological characteristics between it and *Oreocharis mileensis* (W.T.Wang) Mich.Möller & A.Weber. Therefore, *O. mileensis* is likely a duplicate publication of *O. notochlaena*. *O. notochlaena* was published in 1906 by Augustin Abel Hector Léveillé (1906) as a new species under *Didissandra* C.B.Clarke, citing J. Laborde et Émile Bodinier collected specimen number *2684* (Holotype E, E00135155; Isotype P, P03511226) from Tchao-sé (now Zhaosi Village) in Huaxi District, Guizhou Province in 1899. In 1911, H. Léveillé (1911) revised it into *Oreocharis* and changed its scientific name to *Oreocharis notochlaena* H.Lév. Afterward, William Grant Craib (1919) placed it in *Ancylostemon*, with the scientific name changed to *Ancylostemon notochlaenus* (H.Lév. & Vaniot) Craib. *O. mileensis* was first collected in Yunnan in 1906 by F. Ducloux, and the specimens have been stored at the Muséum National d’Histoire Naturelle (P). In 1997, Weitzman et al. (1997) studied this specimen while compiling the *Flora of China* and established *Paraisometrum* with *P. mileensis* as the type species. After the first collection, no botanist collected it in the wild until a century later, when wild populations were discovered in Shilin County, Longlin County, and Xingyi City (Shui 2007; Xu et al. 2009; Gao and Xu 2011). Since the establishment of *Oreocharis* s.l., *O. mileensis* and *O. notochlaena* have been included as two independent species within *Oreocharis* s.l.

## Material and methods

### Materials collection

The materials of *O. villosa, O. wanshanensis*, and *O. brachypoda* were collected from their respective type localities. The materials of *O. notochlaena* come from two populations, one from the type locality (Zhaosi Village, Huaxi District) and the other from Wangyou Town, Huishui County. The materials of *O. mileensis* were collected from Shilin County, Yunnan Province. High-definition photos of plant anatomy were taken during the field survey, and geographic coordinate information was recorded. Meanwhile, healthy leaves were collected from various locations and stored in sealed bags for DNA extraction. The specimens were maintained in the Tree Herbarium of the College of Forestry, Guizhou University (GZAC).

### Morphological comparison

**Fig. 2:**
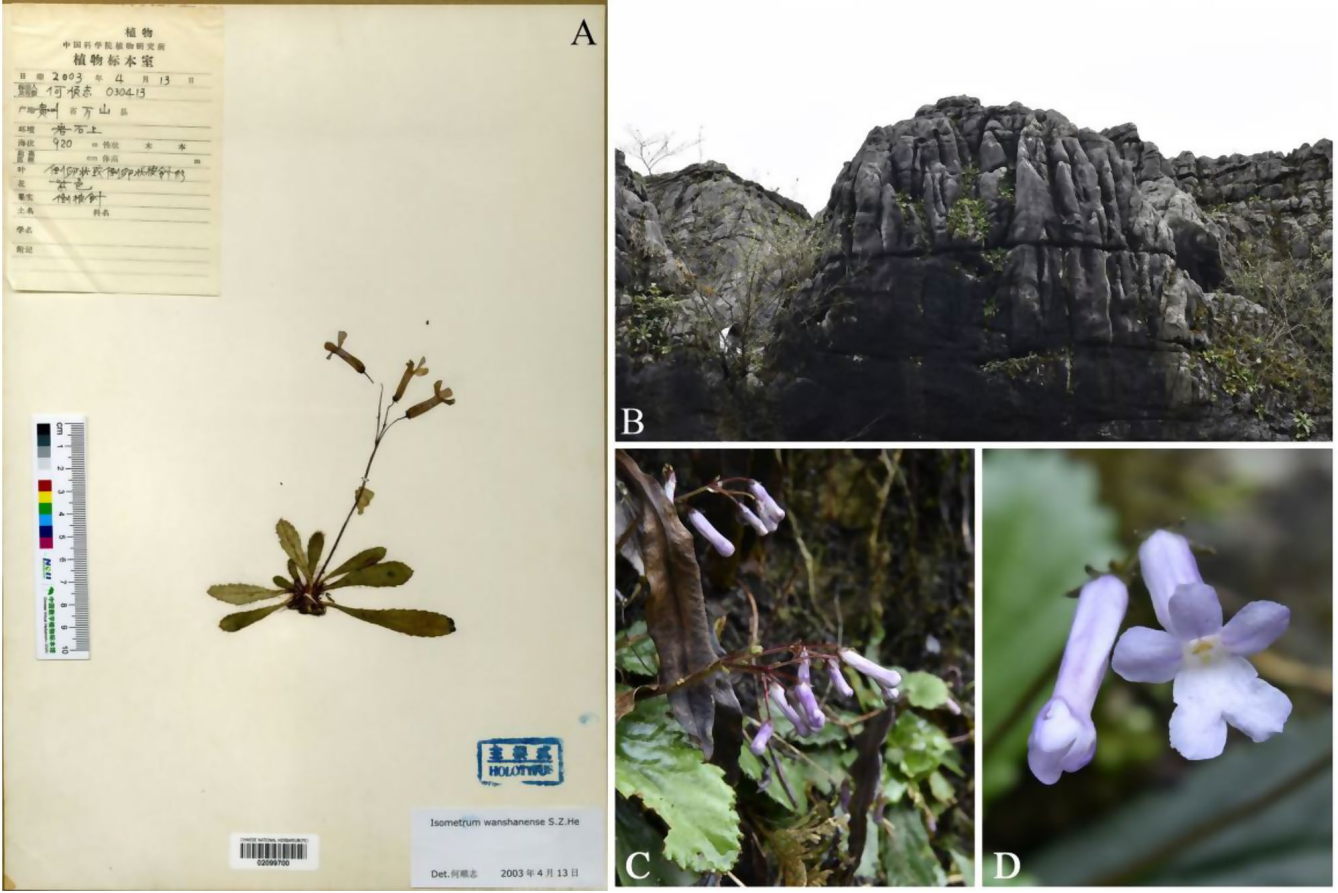
*Oreocharis wanshanensis* (**A** Holotype; **B** habit; **C** flowering plants; **D** frontal view of corolla)

**Fig. 3:**
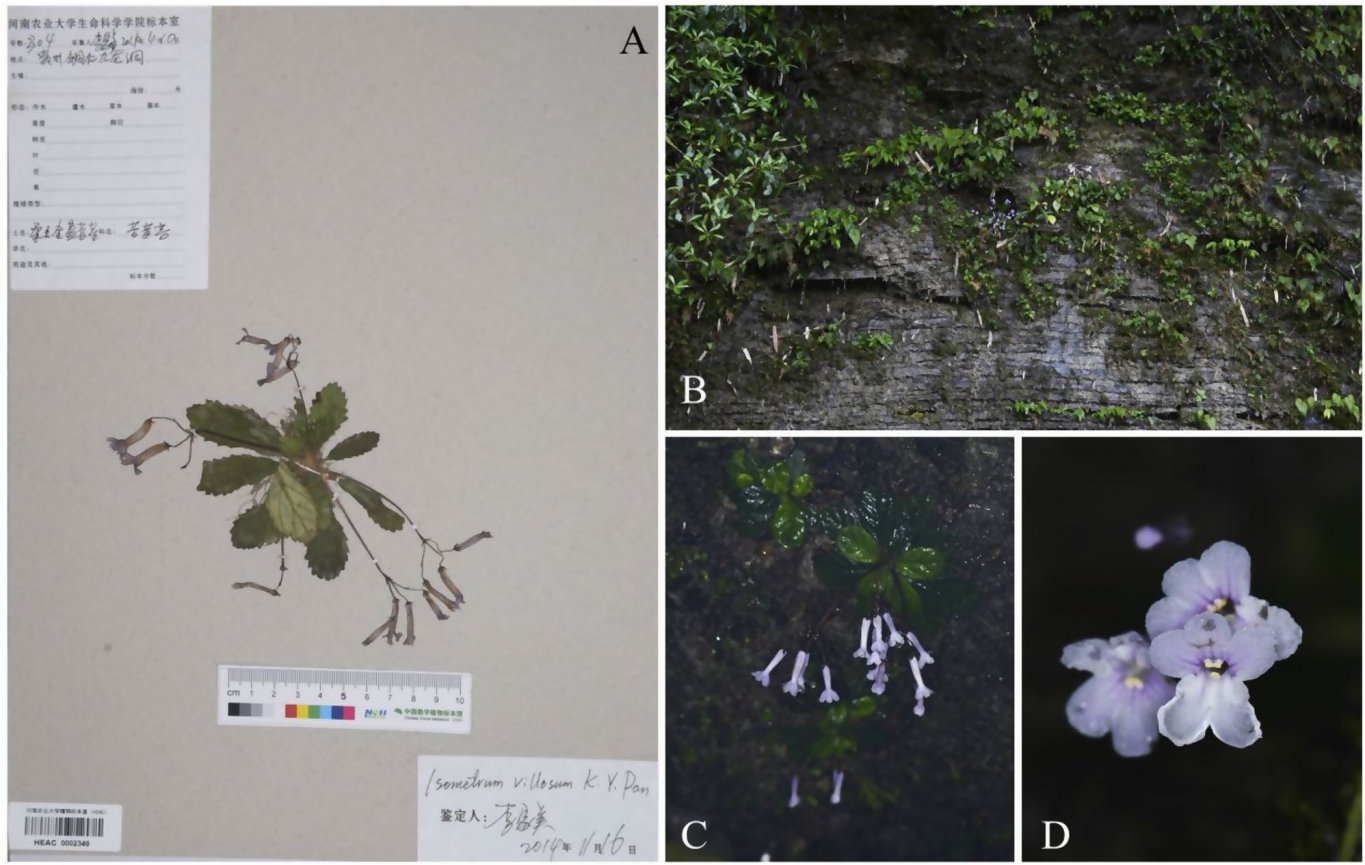
*Oreocharis brachypoda* (**A** Holotype; **B** habit; **C** flowering plants; **D** frontal view of corolla)

**Fig. 4:**
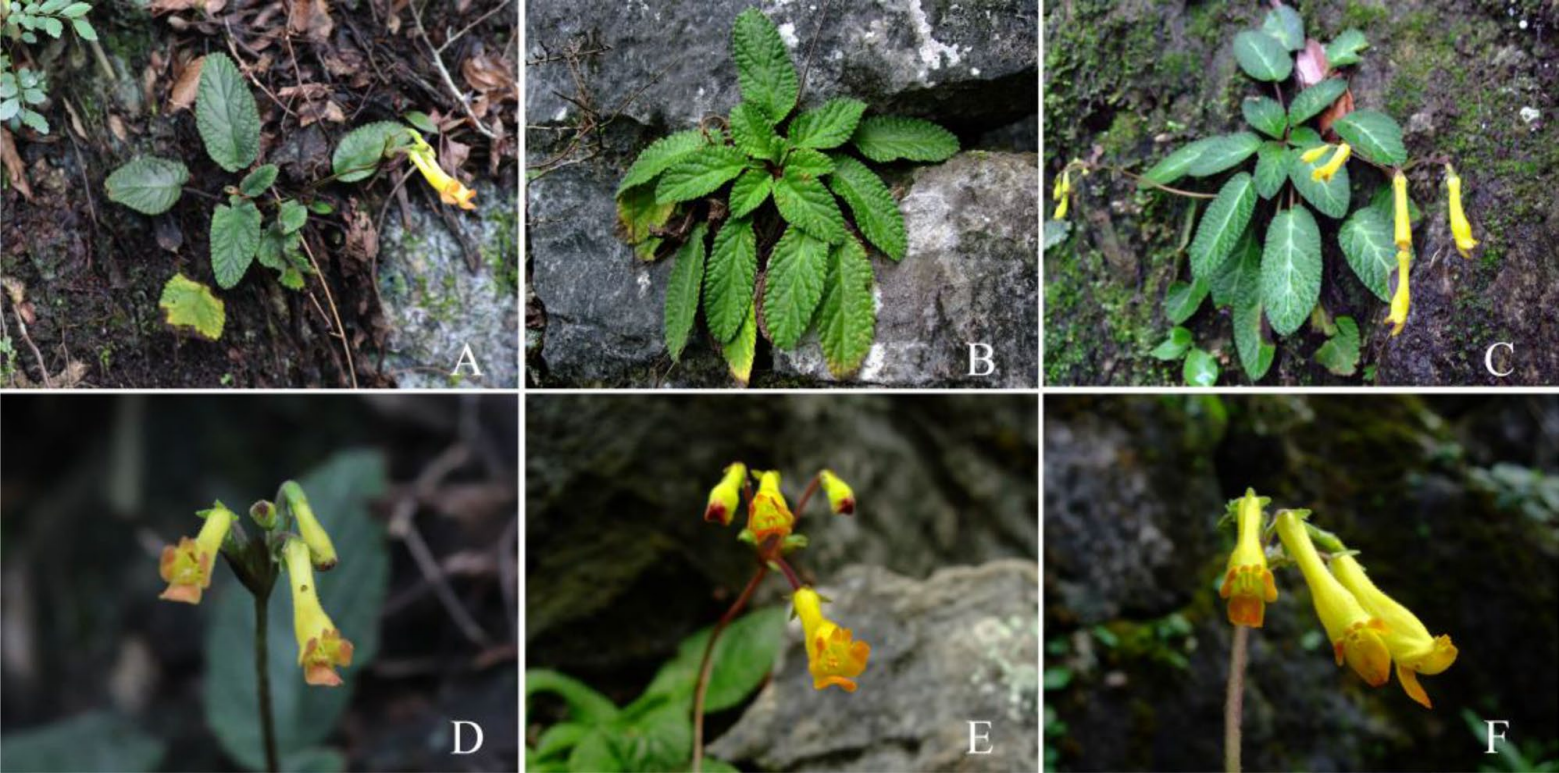
Comparison of populations of *oreocharis notochlaena* and *oreocharis mileensis* (**A **, **D** Huishui population of *O. notochlaena*; **B **, **E** Xingyi population of *O. mileensis*; **C **, **F** Longlin population of *O. mileensis*)

**Fig. 5:**
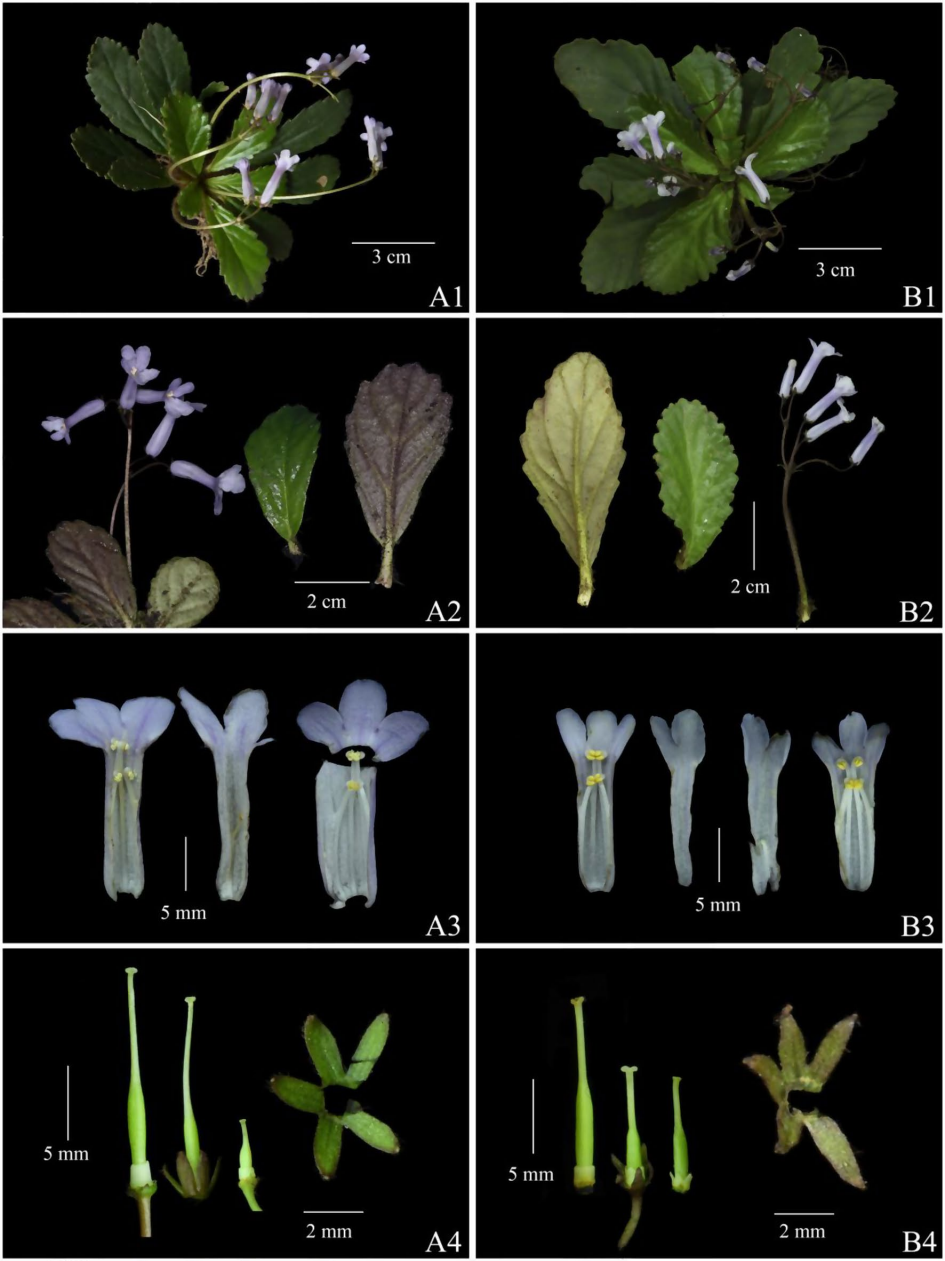
Morphological comparison of *oreocharis brachypoda* and *O. wanshanensis* (**A** *O. brachypoda*; **B** *O. wanshanensis*: 1 plant in flowering; 2 leaves and inflorescences; 3 dissected corolla; 4 calyx and pistil with disk）

**Fig. 6:**
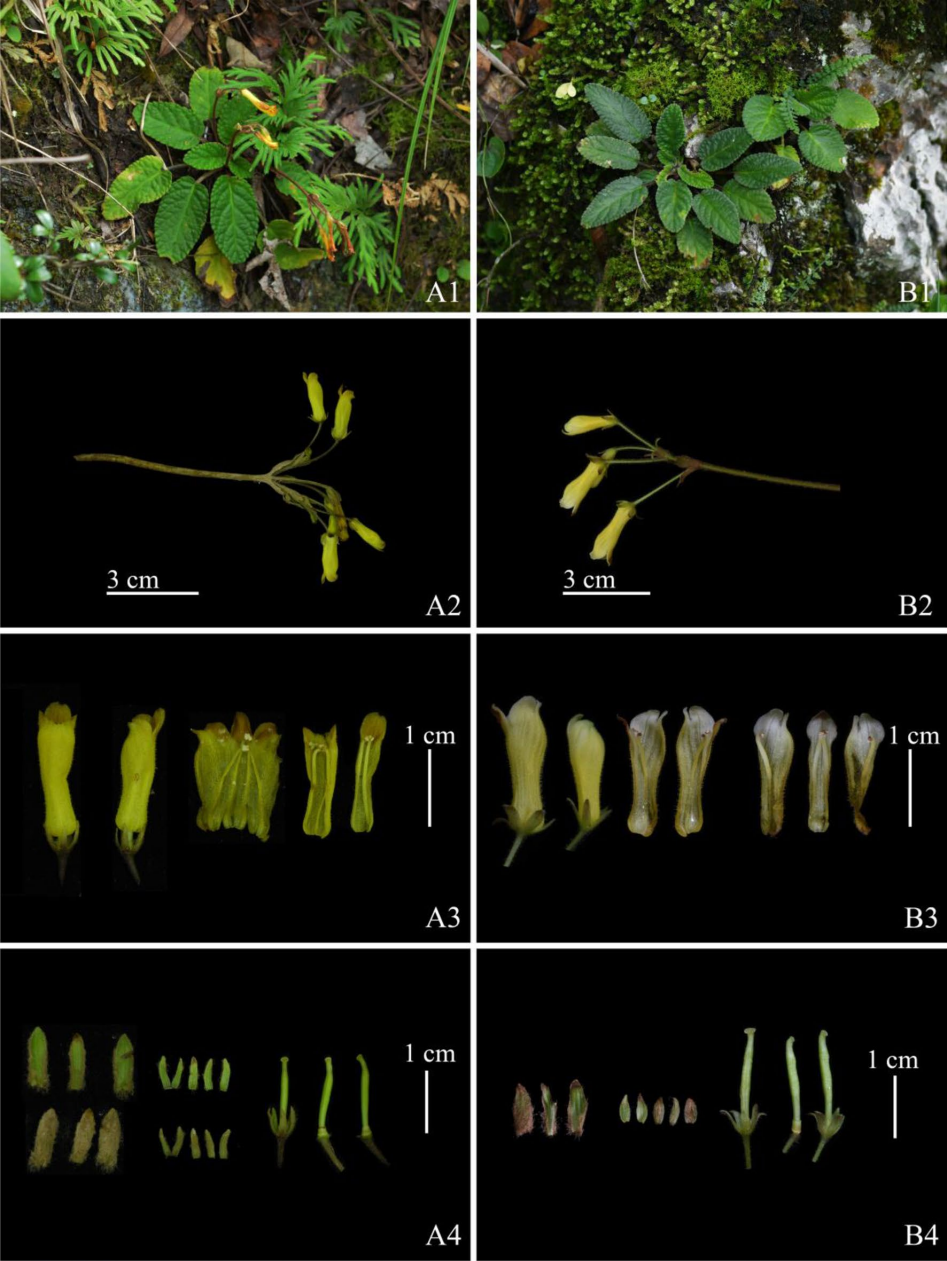
Morphological comparison of *Oreocharis notochlaena* and *oreocharis mileensis* (**A** *O. notochlaena*; **B** *O. mileensis*: 1 plant; 2 inflorescences; 3 dissected corolla; 4 bracts, sepals and pistil)

Detailed comparisons of the species’ morphological characteristics were conducted by examining the original literature. Based on the records of the place of origin, we conducted multiple investigations at the type localities and their surrounding areas of various species (Figs. 2, 3, 4), recording the growth and distribution of the populations, collecting plant specimens, and carefully comparing key morphological characteristics by dissecting live plant materials (Figs. 5, 6). At the same time, we utilized the Chinese Virtual Herbarium (CVH) (http://www.cvh.ac.cn) to examine high-resolution images of type specimens and other specimens from the P, E, HEAC, KUN, GZTM, CSFI, and PE.

### Dna extraction, amplification and sequencing

Total genomic DNA was extracted from silica-gel-dried leaves using the CTAB protocol (Doyle and Doyle 1987). In the phylogenetic analysis of this study, a nuclear ribosomal DNA internal transcribed spacer (ITS) and a chloroplast *trn*L-F intergenic spacer (*trn*L-F) of 68 species were obtained from GenBank, representing the majority of available sequences for *Oreocharis*. In addition, this study collected an additional 23 samples (including six samples of *O. villosa*, three samples of *O. wanshanensis*, three samples of *O. brachypoda*, four samples of *O. notochlaena* from Huaxi District, three samples from Huishui County, and four samples of *O. mileensis* from Shilin County) to obtain DNA sequences. The rest of *O. mileensis* sequences were obtained from Chen et al. (2014). According to previous studies (Möller et al. 2011b; Lv et al. 2022), we selected one species from the *Metapetrocosmea* W.T.Wang and two species from the *Agalmyla* Blume as outgroups, namely *Metapetrocosmea peltata* (Merr. & Chun) W.T.Wang, *Agalmyla biflora* (Elmer) Hilliard & B.L.Burtt, and *Agalmyla clarkei* (Elmer) B.L.Burtt. This study utilized the nuclear ribosomal DNA internal transcribed spacer (ITS) and the chloroplast *trn*L-F intergenic spacer (*trn*L-F) for research. PCR amplification using universal primers for ITS and *trn*L-F sequences (Taberlet et al. 1991; Wendel et al. 1995). The PCR reaction system is 25 L, which includes 2.5 L 10X buffer, 1.0 L 10 mmol/L dNTPs, 1.0 uL of 10 mol/L of each primer, 1.0 L DNA, 0.2 L 5 U/L Taq enzyme, and 25 L ddH2O. PCR conditions were as follows: first 95 °C for 5 min, 1 cycle of 94 °C for 1 min, second 50 °C for 1 min, 72 °C for 50 s, 30 cycles of 95 °C for 1 min, then 50 °C for 1 min, 72 °C for 1 min, and 72 °C for 10 min. 72 °C for 1 min and 72 °C for 10 min. Finally, PCR products were detected by 1% agarose gel electrophoresis, and the qualified samples were sent to Shanghai Sangon Biotech for sequencing. The obtained ITS and *trn*L-F sequences were uploaded to the NCBI database to obtain GenBank accession numbers.

### Phylogenetic analysis

After organizing all the obtained sequences, the nucleotide sequences were aligned using the online version of MAFFT 7 (https://mafft.cbrc.jp/alignment/software/windows.html) (Katoh et al. 2019) and manually trimmed in MEGA 7.0 (Kumar et al. 2016). The Incongruence Length Difference (ILD) test was conducted using the default parameters in PAUP* 4.0a169, which indicated no significant incongruence between ITS and *trn*L-F (*p* > 0.05) (Swofford 2002). The concatenated matrix used in this analysis had a length of 1637 bp (ITS: 744; *trn*L-F: 893), and the concatenation of gene sequences for each species was completed using the Concatenate Sequence module in PhyloSuite v1.2.3 (Zhang et al. 2020). This study is based on Maximum Likelihood (ML) and Bayesian inference (BI) for phylogenetic analysis of individual sequence matrix (ITS/*trn*L-F) and concatenated matrix (ITS-*trn*L-F). Based on the Akaike Information Criteria (AIC) (Akaike 1981), the GTR+I+G and GTR+G were determined using MrModeltest v2.4 (Nylander 2004) as the best-fit models for ITS and *trn*L-F,.respectively. The Maximum Likelihood (ML) analysis was conducted using IQ-TREE v2.3.5 (Nguyen et al. 2015). The ModelFinder in IQ-TREE tested 484 DNA models and selected GTR+F+I+R3 as the best-fit model for the concatenated matrix. Bootstrap consensus values were calculated using 1000 replicates, with all other parameters set to default. Bayesian inference (BI) analysis was conducted using MrBayes v3.2.7 (Ronquist and Huelsenbeck 2003). Two independent Markov Chain Monte Carlo (MCMC) runs with 10,000,000 generations were performed for the analysis. Trees were sampled every 1000 generations. The first 25% of the sampled trees were discarded as burn-in, and the remaining trees were used to build a 50% majority-rule consensus tree. Analyses were run until the average standard deviation of the split frequencies approached 0.01, indicating that two runs converged to a stationary distribution. The best-fit model for the concatenated matrix of Bayesian analysis was calculated as GTR+I+G by MrModeltest v2.4.

## Haplotype analysis

### Haplotype analysis of *O. villosa*, *O. wanshanensis*, and *O. brachypoda*

Twelve ITS sequences and *trn*L-F sequences (6 for *O. villosa*, 3 for *O. wanshanensis*, and 3 for *O. brachypoda*) were selected for haplotype analysis of three closely related species to determine the haplotype types of each species and whether shared haplotypes exist between species. Haplotype reconstruction was performed using DnaSP 6.0, and Haplotype diversity (Hd), Nucleotide diversity (Pi), and Average number of nucleotide differences (K) were calculated (Rozas et al. 2017). Haplotype networks were constructed using Popart 1.7 (Leigh and Bryant 2015).

### Haplotype analysis of *O. notochlaena* and *O. mileensis*

Twenty-six ITS sequences and *trn*L-F sequences of *O. notochlaena* and *O. mileensis* (4 sequences from the Huaxi population of *O. notochlaena*, three sequences from the Huishui population, nine sequences from the Yunnan population of *O. mileensis*, five sequences from the Guangxi population, and five sequences from the Guizhou population) were selected for haplotype analysis in order to determine the haplotype types of each species and whether shared haplotypes exist between species. The software and methods are the same as above.

## Results and discussion

### Morphological research of *O. villosa*, *O. wanshanensis*, and *O. brachypoda*

The main taxonomic morphological characteristics, including corolla, leaf blade, calyx, pistil, and stamen, of *O. brachypoda* and *O. wanshanensis* were compared. On the type specimens (Figs. 2A and 3A), the leaf blade of *O. wanshanensis* is lanceolate. However, observations of multiple field individuals of *O. wanshanensis* revealed a continuous transition between oblanceolate and obovate, which is consistent with the continuous transition in the leaf shape of *O. brachypoda*. During the observation of wild plants, it was also found that there were slight variations in some characteristics. In the protologue of *O. brachypoda*, the disk was described as “many-lobed or entire,” which was more significant in the population of *O. wanshanensis*. Some individuals had parted disks, and the variation range of disks could be summarized as from parted to entire (Fig. 5: A-4, B-4). The pistil of *O. brachypoda* is generally slightly longer than that of *O. wanshanensis*, and this difference is within a reasonable range of variation with a noticeable transition. The variation range of the pistil can be summarized as 8–17 mm in length (Fig. 5: A-4, B-4). Through further detailed comparison (Table 1, Fig. 5), the two are highly consistent in other qualitative characteristics, while there is overlap in quantitative characteristics.

**Table 1:** Morphological comparison of *Oreocharis brachypoda* and *Oreocharis wanshanensis*

Characters	O. brachypoda*	O. brachypoda#	O. wanshanensis*	*O. wanshanensis*#
Leaf shape	obovate	obovate or oblanceolate	oblanceolate	obovate or oblanceolate
Leaf size (cm)	2–5 × 1.5–3. 2	1.5–5 × 1–3. 2	1.5–4 × 0.5–1.6	1.5–5.5 × 0.8–3.5
Petiole Length(mm)	0–2	0–11	0.4–14	0–12
Flower number	1–4	1–4	1–4	1–5
Peduncle Length(cm)	4–6.5	4–6	4–7	4–6
Calyx shape	narrowly triangular	narrowly triangular	lanceolate	narrowly triangular
Calyx Length(mm)	3	3	2.5	3
Corolla color	white to light purple	white to light purple	purple	white to light purple
Corolla Length(cm)	2. 2–2. 5	1.8–2.3	2	1.7–2.2
Corolla tube Length（mm）	15–18	14–17	15	14–18
Stamens Length(mm)	6–8	5–9	4–5	4–9
Anther connection	coherent in pairs	coherent in pairs, occasionally	coherent in pairs	coherent in pairs, occasionally
Pistil Length（mm）	17	11–17	12–14	8–16

Note: * represents description based on the original literature; # represents description based on examination of specimens and observations of wild individuals from the type locality

Based on the research of the original literature, *O. villosa* is undoubtedly a close relative species of *O. brachypoda* and *O. wanshanensis*. In the descriptions of He (2006) and Li and Li (2015), *O. villosa* is different from the above two in that its leaves sessile (vs. leaves stipitate, 0.4–1.4 cm long), leaf blades obovate-oblong (vs. leaf blades oblanceolate), cymes 2–3 branches, 2–3 per plant, each 8–10-flowered (vs. cymes unbranched, 1–4 per plant, each 1–4-flowered), anther thecae divergent (vs. anther thecae confluent), style shorter than ovary (vs. style and ovary nearly equal in length). Through comparison of field populations, it was found that the characteristics of petiole length and leaf shape are unstable, showing variation among populations. However, the number of flowers per inflorescence, whether the anther thecae are confluent or not, and the length of the style and ovary of *O. villosa* differ from those of *O. brachypoda* and *O. wanshanensis*.

### Morphological research of *O. notochlaena* and *O. mileensis*

**Fig. 7:**
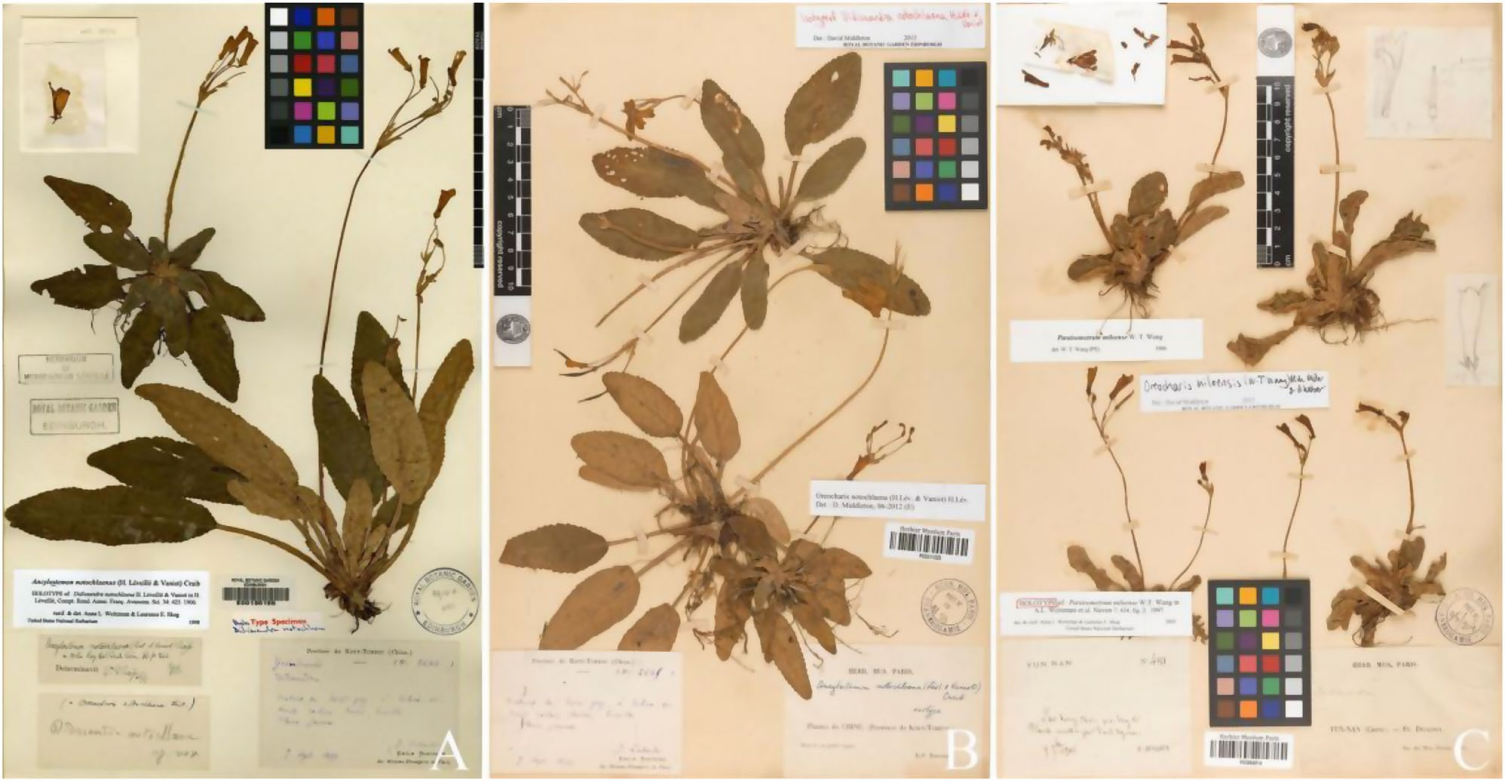
Type specimens comparison of Oreocharis notochlaena and *O. mileensis* (**A** Holotype of *O. notochlaena* **B** Isotype of *O. notochlaena* **C** Holotype of *O. mileensis*)

In the supplemental descriptions of *O. notochlaena* and the original description of *O. mileensis* (Weitzman et al. 1997; Wang et al. 1998; Li and Wang 2005), the abaxial surface of the leaves, bracts, and peduncles of *O. notochlaena* was described as pannose, while *O. mileensis* was described as lanuginous. Based on both herbarium specimens and field observations of living plants, they should all be pannose. For the characteristics of the limb, the former was described as the upper lip extremely short, slightly concave, lower lip 3-lobed, central lobe much longer than lateral lobes, and the latter was described as the upper lip unequal 4-lobed, lobes triangular, central 2 lobes smaller, lateral 2 lobes larger, lower lip undivided. The characteristics presented in the different descriptions of the limb are similar, but the description of *O. notochlaena* was more accurate. Comparing the type specimens of the two (Fig. 7), the leaves of *O. notochlaena* are lanceolate to oblong-elliptical. Observations of leaf morphological characteristics in multiple field individuals revealed a transition in leaf shape between lanceolate and oblong-elliptical, consistent with the leaf shape of *O. mileensis.* In different populations of *O. mileensis*, the leaf shape and veins vary slightly depending on the habitat. The leaf shape can be elliptical or oblong-elliptical, and the veins can be distinct. The bracts are lanceolate or narrowly ovate. These characteristics vary within a reasonable range, and there is continuous transition between different populations. Detailed comparisons of plants in the field (Table 2, Figs. 6 and 4) show no morphological characteristics that can effectively distinguish between *O. notochlaena* and *O. mileensis*.

**Table 2:** Morphological comparison of *Oreocharis notochlaena* and *Oreocharis mileensis*

Characters	O. notochlaena	O. mileensis
Leaf shape	lanceolate or oblong-elliptical	elliptical or oblong-elliptical
Leaf size (cm)	3–12 × 1.5–3.2	2.5–8.1 × 1.2–3.0
Leaf margin	crenulate	crenulate
Inflorescence number	1–3	1–2
Flower number	3–8	3–6
Peduncle Length(cm)	densely pale brownish pubescent	densely brownish pubescent
Pedicel	glandular-pubescent and pubescent	glandular-pubescent and pubescent
Bract	lanceolate-oblong, abaxially pale brownish pannose, adaxially glabrous	linear-lanceolate, abaxially pale brownish lanuginous, adaxially glabrous
Calyx shape	linear-lanceolate	linear-lanceolate
Corolla color	orange-yellow	yellow
Corolla Length (cm)	1.5–2.0	1.6–1.8
Corolla tube Length (mm)	1.3–1.4	1.1–1.5
Corolla mouth diameter (cm)	3.7–4.5	3.5–5
Limb	upper lip 4-lobed, lobes triangular, lower lip undivided, oval	upper lip 4-lobed, lobes triangular, lower lip undivided, deltoid-ovate
Anther connection	coherent in pairs	coherent in pairs
Pistil	ovary glabrous, style glandular-pubescent, stigma flat-capitate	ovary glabrous, style glandular-pubescent, stigma flat-capitate

### Phylogenetic analysis

**Fig. 8:**
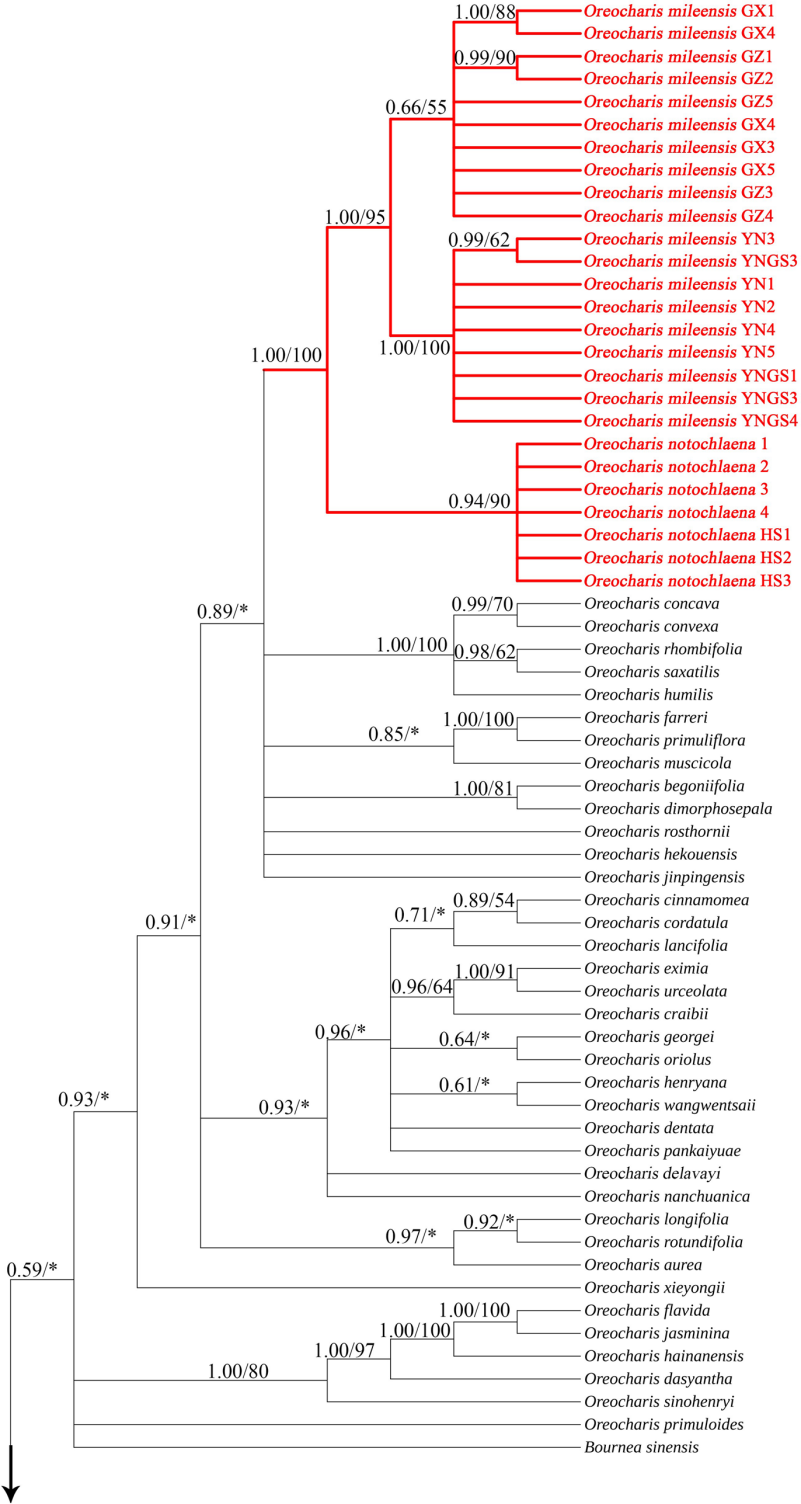

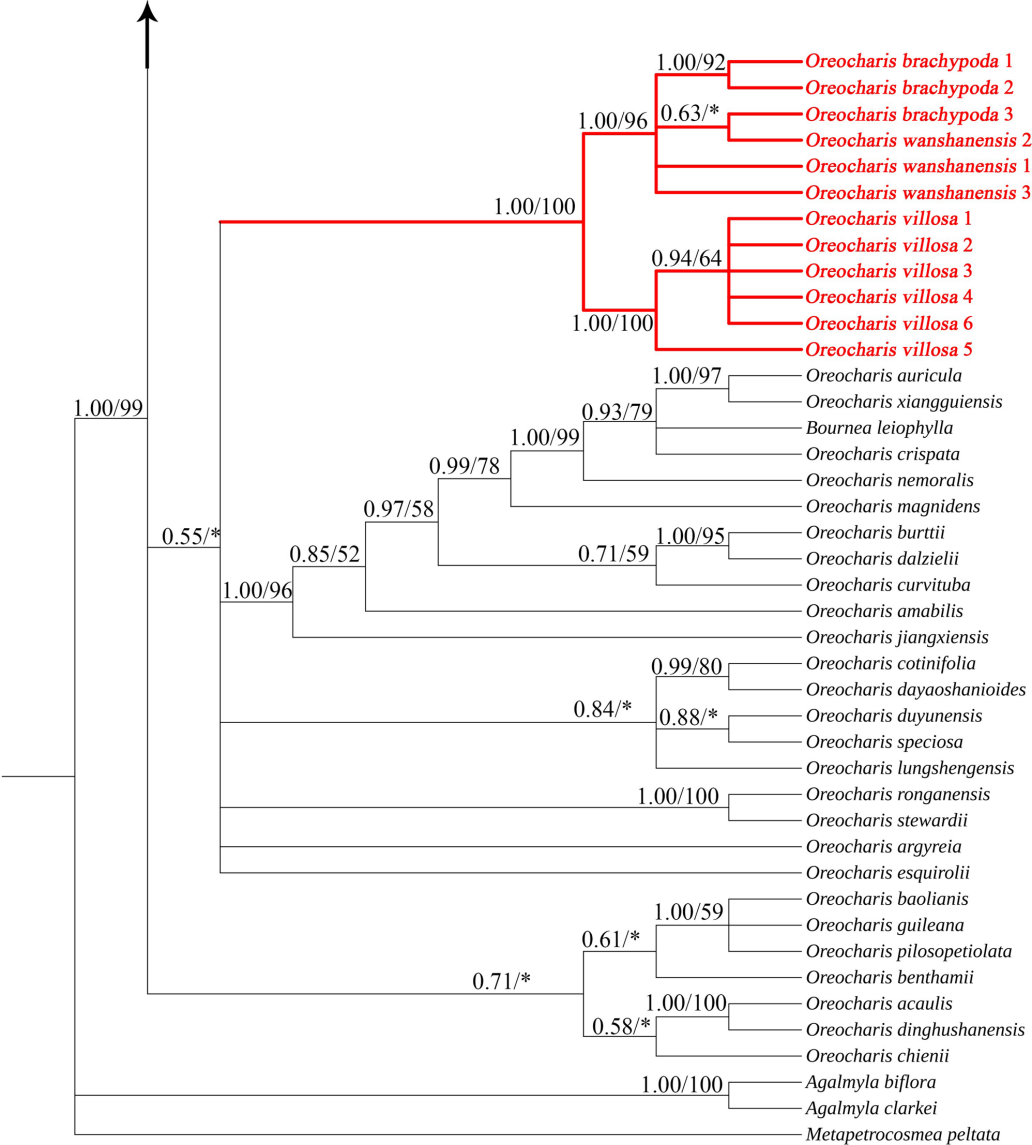
Bayesian 50% majority-rule consensus tree inferred from combined nrITS and *trn*L-F sequences data. Values above branches are bayesian posterior probabilities ( > 0.5)/maximum likelihood bootstrap percentages ( > 50). “*” denotes branches with < 50% bootstrap support

The ML and BI tree constructed from the concatenated matrix of ITS and *trn*L-F had a similar topology but differed in branch support (Figs. S1 and S2). Thus, the branch support values of the results of both reconstructions can be summarized on a Bayesian consensus tree. The BI tree constructed based on 106 concatenated sequences is shown in Fig. 8, with posterior probabilities for BI and bootstrap support values for ML annotated above each branch (PP < 0.5 and BS < 50% are not shown). The phylogenetic analysis shows that, apart from the outgroups, 103 samples formed a monophyletic group with strong support (PP = 1, BS = 99%). The topology of other branches is generally consistent with previous studies (Ling et al. 2022; Lv et al. 2022; Hu et al. 2023). However, the current phylogenetic analysis does not fully resolve the phylogenetic relationships within *Oreocharis* s.l. In the phylogenetic tree, 12 samples of *O. villosa*, *O. wanshanensis*, and *O. brachypoda* formed a subclade with the highest support (PP = 1, BS = 100%). At the same time, within the subclade, *O. wanshanensis* and *O. brachypoda* were nested together to form a well-supported clade (PP = 1, BS = 96%). As a closely related species of *O. wanshanensis* and *O. brachypoda*, *O. villosa* formed a close clade with these two species in the phylogenetic tree. A total of 26 samples of *O. notochlaena* and *O. mileensis* formed a clade with the highest support (PP = 1, BS = 100%). Among them, *O. notochlaena* from two populations were nested in a clade (PP = 0.94, BS = 90%). *O. mileensis* from Guizhou and Guangxi were mixed to form a clade, while samples from Yunnan were most similar and formed a highly supported clade in the phylogenetic tree (PP = 1, BS = 100%).

**Fig. 9:**
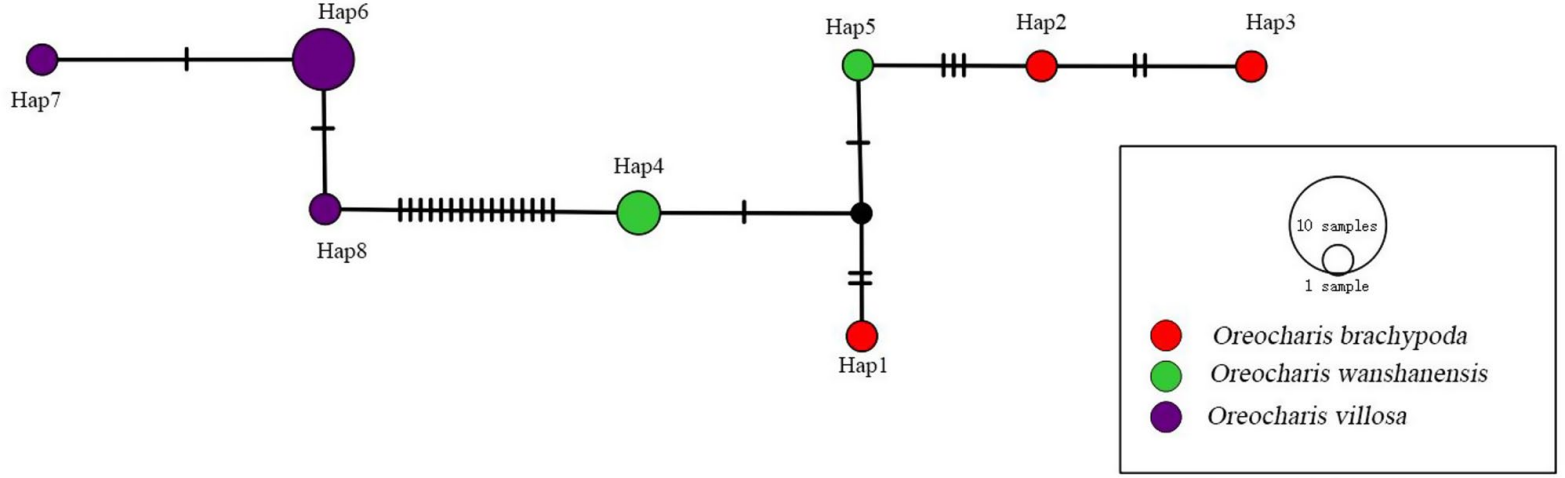
TCS-derived network based on the ITS sequences

### Haplotype diversity, nucleotide diversity, and haplotype network analysis of *O. villosa*, *O. wanshanensis*, and *O. brachypoda*

**Fig. 10:**
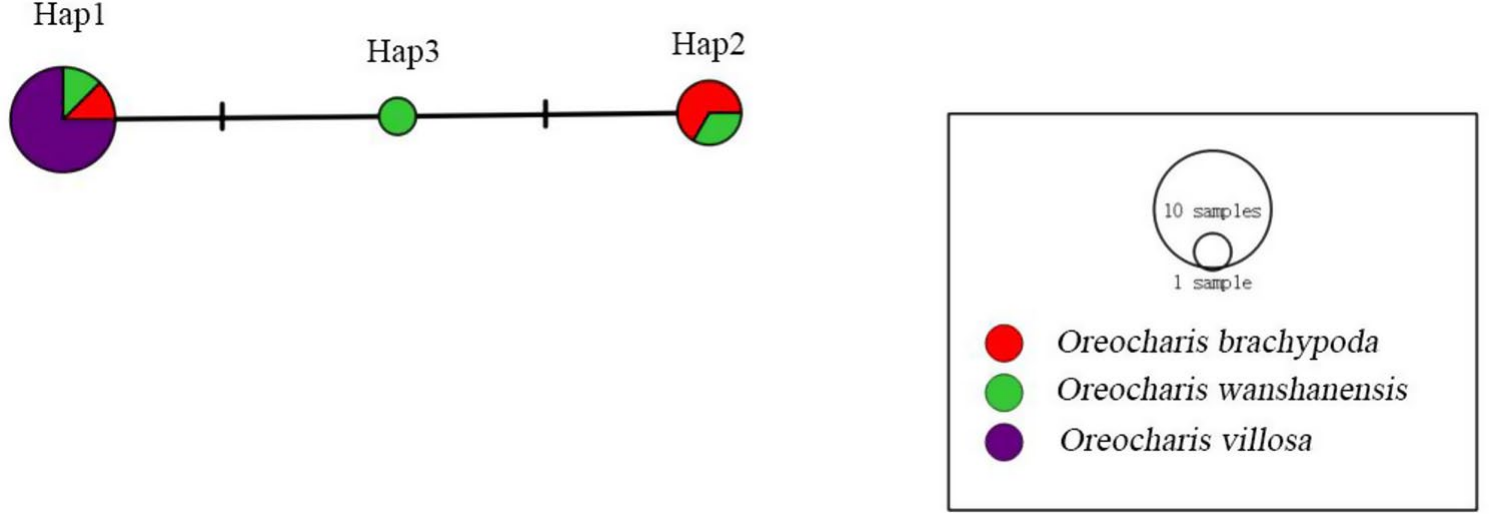
TCS-derived network based on the *trn*L-F sequences

Twenty-three polymorphic sites were found in the 12 ITS sequences, including 21 parsimony informative sites (P) and two singleton variable sites (S). The dataset includes eight haplotypes, with haplotype diversity (Hd) and nucleotide diversity (Pi) of 0.894 and 0.016, respectively. The average number of nucleotide differences (K) is 11.167 (Table 3). The TCS-derived network of ITS sequences shows that the haplotypes between *O. brachypoda* and *O. wanshanensis* are directly connected with a small number of mutation steps. However, the haplotype differences between *O. wanshanensis* and *O. villosa* require 16 nucleobase substitutions, and they do not share any haplotypes with each other, indicating that there may be natural genetic isolation between them (Fig. 9). A total of 2 polymorphic sites were found in the 12 *trn*L-F sequences, including two parsimony informative sites (P) and no singleton variable sites (S). The dataset includes three haplotypes, with haplotype diversity (Hd) and nucleotide diversity (Pi) of 0.530 and 0.018, respectively. The average number of nucleotide differences (K) is 0.894 (Table 4). The TCS-derived network of *trn*L-F sequences shows direct connections with minimal mutational steps between the three and the presence of shared haplotypes (Fig. 10).

**Table 3:** Polymorphic sites and diversity of DNA sequences of ITS sequences

Singleton variable sites (S)	Parsimony informative sites (P)	Haplotype diversity (Hd)	Nucleotide diversity (Pi)	Average number of nucleotide differences (K)
2	21	0.894	0.016	11.167

**Table 4:** Polymorphic sites and diversity of DNA sequences of *trn*l-f sequences

Singleton variable sites (S)	Parsimony informative sites (P)	Haplotype diversity (Hd)	Nucleotide diversity (Pi)	Average number of nucleotide differences (K)
0	2	0.530	0.018	0.894

### Haplotype diversity, nucleotide diversity, and haplotype network analysis of *O. notochlaena* and *O. mileensis*

**Fig. 11:**
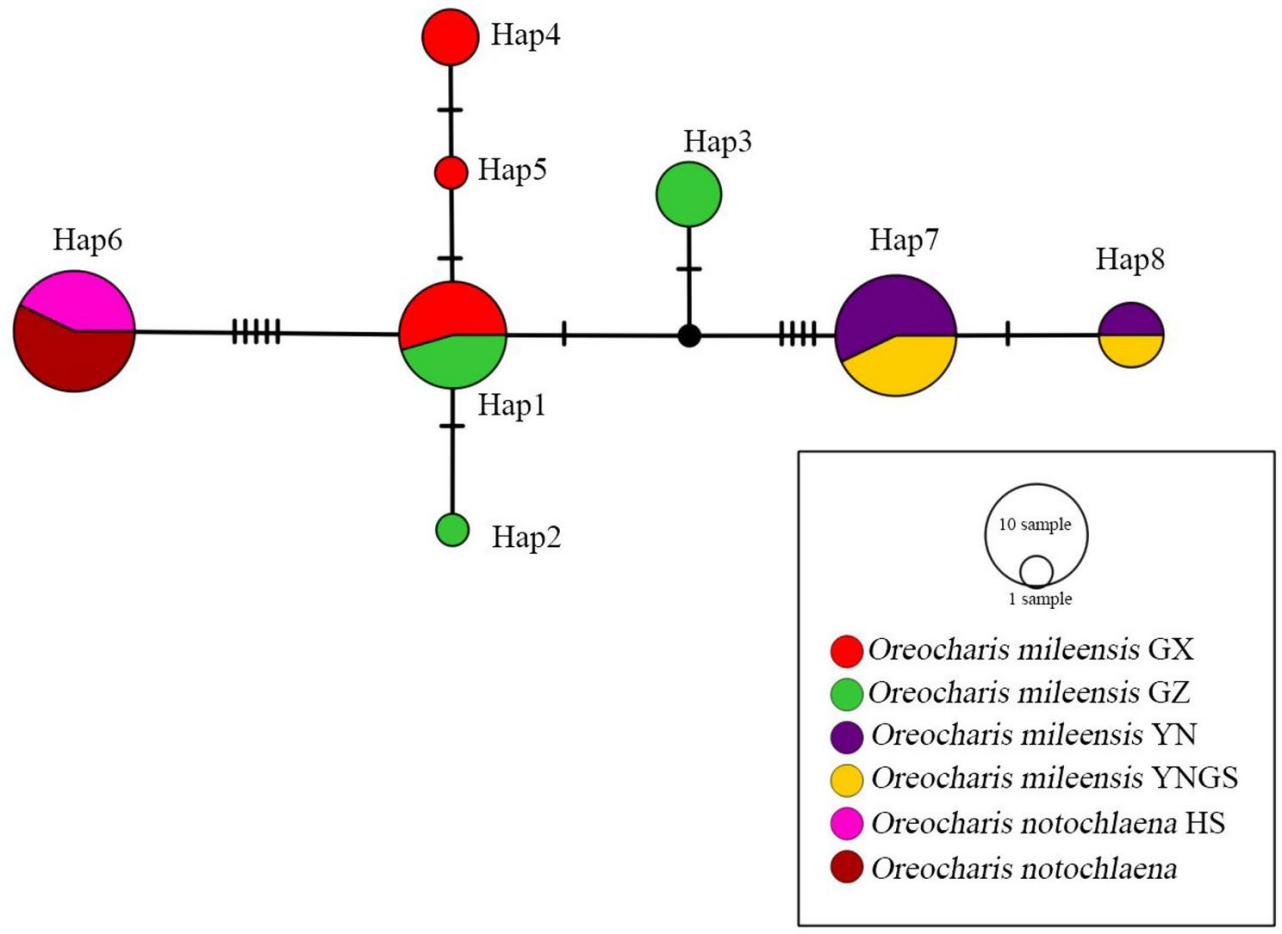
TCS-derived network based on the ITS sequences

Fourteen polymorphic sites were found in the 26 ITS sequences, including 13 parsimony informative sites (P) and one singleton variable site (S). The dataset includes eight haplotypes, with haplotype diversity (Hd) and nucleotide diversity (Pi) of 0.81 and 0.00755, respectively. The average number of nucleotide differences (K) is 4.554 (Table 5). The TCS-derived network of ITS sequences shows that there are shared haplotypes between two populations of *O. notochlaena*, and it is directly connected to *O. mileensis* from Xingyi City, Guizhou, and Longlin County, Guangxi, with a small number of mutation steps. Samples from Yunnan do not have a shared haplotype with other populations (Fig. 11). The 26 *trn*L-F sequences contain only one haplotype and no polymorphic sites. The haplotype diversity (Hd) and nucleotide diversity (Pi) are both 0.

**Table 5:** Polymorphic sites and diversity of DNA sequences of ITS sequences

Singleton variable sites (S)	Parsimony informative sites (P)	Haplotype diversity (Hd)	Nucleotide diversity (Pi)	Average number of nucleotide differences (K)
1	13	0.81	0.00755	4.554

In summary, compared to the ITS sequence, the *trn*L-F sequence has fewer singleton variable sites, parsimony informative sites, haplotype diversity, and average number of nucleotide differences. As a maternally inherited chloroplast fragment, *trn*L-F is more conservative and may be used to better trace the evolutionary history of plants. From the constructed TCS-derived network, it can be observed that there are little sequence differences between *O. brachypoda* and *O. wanshanensis*, as well as little sequence differences between *O. notochlaena* and *O. mileensis*, further corroborating the results of morphological research and phylogenetic analysis.

## Taxonomic treatment

*Oreocharis villosa* **var. wanshanensis** (S.Z.He) X.X.Bai & F.Wen, **comb. & stat. nov.:** *Oreocharis wanshanensis* (S.Z.He) Mich.Möller & A.Weber in Phytotaxa 23(1): 28. 2011. *——Isometrum wanshanense* S.Z.He in Acta Phytotax Sin. 44(4): 454. 2006. **TYPE:** China. Guizhou Province: Tongren City, Wanshan, alt. 970 m, Karst hills, April 13 2003, *S.Z.He* 030413 (holotype, PE02099700!; isotypes, HGCM0073029!, PE02099702!, PE02099701!).

=*Oreocharis brachypodus* J.M.Li & Zhi M.Li in Phytotaxa 204(4): 296. 2015. **syn. nov.**

Type: China. Guizhou: in the vicinity of Tongren City, on relatively cool rocks and very steep banks of cool, clammy soil that grows a fine film of moss, alt. 1300 m, 9 April 2014, *Jia-Mei Li* 2304 (holotype: HEAC0002349!); ibid. *Jia-Mei Li* 2305 (paratype: HEAC0002350!).

**Chinese Vernacular name:**万山金盏苣苔 (Wàn Shān Jīn Zhǎn Jù Tái)

**Difference from the original variant:** cymes unbranched, 1–4-flowered, anther thecae confluent, style and ovary nearly equal in length.

**Fig. 12:**
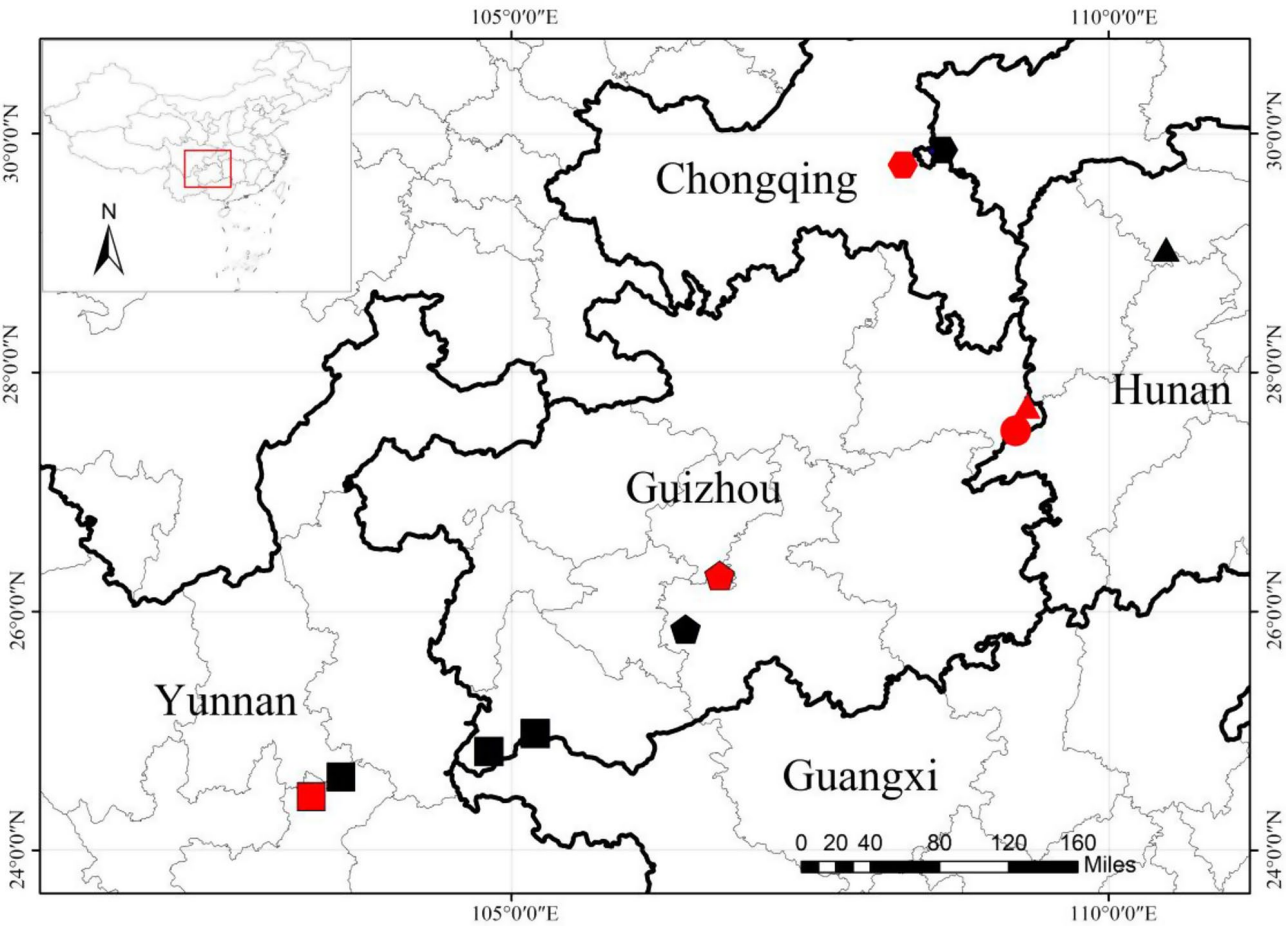
Geographical distribution of *oreocharis wanshanensis*, *O. brachypoda*, *O. villosa*, *O. notochlaena* and *O. mileensis*. Notes: the circle represents the distribution localities of *O. wanshanensis*, the triangle represents the distribution localities of *O. brachypoda*, the hexagon represents the distribution localities of *O. villosa*, the pentagon represents the distribution localities of *O. notochlaena*, and the square represents the distribution localities of *O. mileensis*. The red represents the type locality of the species. Base map sourced from the National platform for common GeoSpatial information services (https://www.Tianditu.gov.cn/), map No. GS(2024)0650

**Distribution and habitat:**Wanshan District and Bijiang District of Tongren City, Guizhou Province, are also distributed in Hunan Province (Fig. 12). Growing on rocks in shady valleys at an altitude of 270–1300 m.

**Additional specimens examined: Guizhou Province:** Wanshan District, alt 836 m, 2 Nov 2013, *Cuiyuan Feng FCY2013025* (PE); Wanshan District, alt 845 m, 29 Mar 2022, *Xinxiang Bai BXX20220329* (GZAC); Bijiang District, alt 465 m, 30 Mar 2022, *Xinxiang Bai BXX20220330* (GZAC); **Hunan Province:** Zhangjiajie City, alt 270 m, May 2011, *Xunlin Yu 1105010* (CSFI); Zhangjiajie City, alt 400 m, 13 Apr 2015, *Hui Zhou & Dasong Zhou 15041301* (CSFI).

*Oreocharis notochlaena* H.Lév. in Repert. Spec. Nov. Regni Veg. 9: 330. 1911. *——Didissandra notochlaena* H.Lév. & Vaniot in Compt. Rend. Assoc. France. 34: 425. 1906. *——Didymocarpus notochlaena* (H.Lév. & Vaniot) H.Lév. 1. c. 428. *——Ancylostemon notochlaenus* (H. Lév. & Vaniot) Craib In: Notes Roy. Bot. Gard. Edinburgh 11: 266. 1919. **Type:** CHINA. Kouy-Tchéou (Guizhou): Tsin-gay, Tchao-sé, 7 Septermber 1899, *E.Bodinier 2684* (Holotype E, E00135155; Isotype P, P03511226).

**=Oreocharis mileensis** (W. T. Wang) Mich. Möller & A.Weber **syn. nov.** in Phytotaxa 23(1): 23. 2011.——*Paraisometrum mileense* W.T.Wang in Novon 7(4): 434. 1998. Type: CHINA. Yunnan: Mile Xian, Lau-kouy chan, 9 Septermber 1906, *F. Ducloux 4515* (Holotype: P03884814).

**Chinese Vernacular name:** 贵州直瓣苣苔 (Guì Zhōu Zhí Bàn Jù Tái)

**Distribution and habitat:** Huaxi District, Huishui County, Xingyi City, Guizhou Province, as well as Yunnan Province and Guangxi Zhuang Autonomous Region (Fig. 12). On the stone wall under the limestone mountain forest, at an altitude of 1000–1200 m.

**Additional specimens examined:** Guizhou Province: Huaxi District, 16 Nov 1985, *Deyuan Chen & Jiaqi Wu 1173* (PE); Mile City, 23 Dec 2016, alt 1908 m, *Jie Cai et al. 16CS11795* (KUN); Shilin County, 25 Sep 2013, alt 2100 m, *Cheng Liu & Xiaojian Hu 13CS6692* (KUN); Shilin County, 2 Sep 2011, *Jiamei Li 4278* (HEAC); Shilin County, 6 Dec 2017, alt 1912 m, *Lei Cai KLBCL025* (KUN).

## Conclusion

In this study, based on morphology, geographic distribution patterns, molecular phylogeny, and haplotype networks, the findings on two taxonomic issues are as follows: First, *O. brachypoda* is a duplicate publication of *O. wanshanensis*, and morphologically, it is distinguished from *O. villosa* by cymes unbranched, 1–4-flowered, anther thecae confluent, style and ovary nearly equal in length. Second, *O. mileensis* is a repeated publication of *O. notochlaena*. According to the regulations and suggestions of the 2018 *International Code of Nomenclature for Algae, Fungi, and Plants (Shenzhen Code)* (Turland et al. 2018), it is proposed to treat *O. wanshanensis* as a variant of *O. villosa* and to treat *O. brachypoda* as a synonym of *O. wanshanensis*. At the same time, *O. mileensis* should be treated as a synonym of *O. notochlaena*. This study clarified the relationships between five species in *Oreocharis* s.l., reasonably classified the species, and provided a scientific basis for effective protection and exploitation of these species.

## Electronic supplementary material

Below is the link to the electronic supplementary material.

Supplementary Material 1

## Data Availability

All data generated and analyzed during this study are included in this published article and its Additional files.
