# Supplementary material for: Two new synonyms and a name at new rank of Oreocharis Benth. (Gesneriaceae) in China
Source: Bot Stud. 2025 Dec 16;66:40. doi: 10.1186/s40529-025-00485-9 (PMC12708486; doi:10.1186/s40529-025-00485-9)
Supplement: Supplementary file 1 — Supplementary Material 1 [file 40529_2025_485_MOESM1_ESM.docx]

**Additional file**

Additional file 1: Fig. S1. Phylogeny based on BI analysis of combined nrITS and *trn*L-F sequences data.

Additional file 2: Fig. S2. Phylogeny based on the ML analysis of combined nrITS and *trn*L-F sequences data.

Additional file 3: Appendix S1. The NCBI accessions for DNA sequences (ITS and *trn*L-F) used in this study.
